# *Trypanosoma janseni* n. sp. (Trypanosomatida: Trypanosomatidae) isolated from *Didelphis aurita* (Mammalia: Didelphidae) in the Atlantic Rainforest of Rio de Janeiro, Brazil: integrative taxonomy and phylogeography within the *Trypanosoma cruzi* clade

**DOI:** 10.1590/0074-02760170297

**Published:** 2018-01

**Authors:** Camila Madeira Tavares Lopes, Rubem Figueiredo Sadok Menna-Barreto, Márcio Galvão Pavan, Mirian Cláudia De Souza Pereira, André Luiz R Roque

**Affiliations:** 1 Fundação Oswaldo Cruz-Fiocruz, Instituto Oswaldo Cruz, Laboratório de Biologia de Tripanosomatídeos, Rio de Janeiro, RJ, Brasil; 2 Fundação Oswaldo Cruz-Fiocruz, Instituto Oswaldo Cruz, Laboratório de Biologia Celular, Rio de Janeiro, RJ, Brasil; 3 Fundação Oswaldo Cruz-Fiocruz, Instituto Oswaldo Cruz, Laboratório de Mosquitos Transmissores de Hematozoários, Rio de Janeiro, RJ, Brasil; 4 Fundação Oswaldo Cruz-Fiocruz, Instituto Oswaldo Cruz, Laboratório de Ultraestrutura Celular, Rio de Janeiro, RJ, Brasil

**Keywords:** Trypanosoma janseni, Didelphis aurita, biological characterisation, integrative taxonomy, phylogeography

## Abstract

**BACKGROUND:**

*Didelphis* spp. are a South American marsupial species that are among the most ancient hosts for the *Trypanosoma* spp.

**OBJECTIVES:**

We characterise a new species (*Trypanosoma janseni* n. sp.) isolated from the spleen and liver tissues of *Didelphis aurita* in the Atlantic Rainforest of Rio de Janeiro, Brazil.

**METHODS:**

The parasites were isolated and a growth curve was performed in NNN and Schneider's media containing 10% foetal bovine serum. Parasite morphology was evaluated via light microscopy on Giemsa-stained culture smears, as well as scanning and transmission electron microscopy. Molecular taxonomy was based on a partial region (737-bp) of the small subunit (18S) ribosomal RNA gene and 708 bp of the nuclear marker, glycosomal glyceraldehyde-3-phosphate dehydrogenase (gGAPDH) genes. Maximum likelihood and Bayesian inference methods were used to perform a species coalescent analysis and to generate individual and concatenated gene trees. Divergence times among species that belong to the *T. cruzi* clade were also inferred.

**FINDINGS:**

*In vitro* growth curves demonstrated a very short log phase, achieving a maximum growth rate at day 3 followed by a sharp decline. Only epimastigote forms were observed under light and scanning microscopy. Transmission electron microscopy analysis showed structures typical to *Trypanosoma* spp., except one structure that presented as single-membraned, usually grouped in stacks of three or four. Phylogeography analyses confirmed the distinct species status of *T. janseni* n. sp. within the *T. cruzi* clade. *Trypanosoma janseni* n. sp. clusters with *T. wauwau* in a well-supported clade, which is exclusive and monophyletic. The separation of the South American *T. wauwau* + *T. janseni* coincides with the separation of the Southern Super Continent.

**CONCLUSIONS:**

This clade is a sister group of the trypanosomes found in Australian marsupials and its discovery sheds light on the initial diversification process based on what we currently know about the *T. cruzi* clade.

Trypanosomes are obligate protozoan parasites that infect all vertebrate classes worldwide. Their life-cycle usually alternates between vertebrate hosts and a variety of sanguinivorous invertebrate hosts that act as vectors. There are a variety of *Trypanosoma* species, but only some of them represent a severe public health and economic challenge. These include *Trypanosoma cruzi*, which is responsible for Chagas disease in South America and other parts of the world, and *T. brucei*, responsible for human and animal African trypanosomiasis.

*Trypanosoma* species from fish and anurans are grouped into the aquatic clade, while the species from the terrestrial clade is divided into two non-taxonomic groups, Salivaria and Stercoraria, according to the parasites' respective mode of transmission. Species from the Salivaria group are cyclically transmitted via salivary inoculation by tsetse flies (*Glossina* spp., Diptera) in Africa, while some are mechanically transmitted by other vectors on other continents, such as the *Tabanus* spp. and *Stomoxys* spp. The Salivaria group comprises five subgenera: *Tejeraia*, *Trypanozoon*, *Nannomonas*, *Duttonella* and *Pycnomonas*. Species from the Stercoraria group are transmitted via a contamination pathway, whereby parasites are eliminated in the faecal contents of different sanguinivorous insects (triatomines and others). This group comprises three subgenera: *Megatrypanum*, *Herpetosoma* and *Schizotrypanum* (Lima et al. 2015).

It is proposed that the genus *Trypanosoma* contains a monophyletic group of organisms that evolved from a common African ancestor. Two main hypotheses were proposed for the origin and dispersion of the species in the *T. cruzi* clade. The first, the vicariant southern supercontinent hypothesis, is based on the continental drift as a source of separation between the ancestral *Trypanosoma* populations, which may have caused population divergence and speciation (Hamilton et al. 2012). In this sense, the separation of Africa and the Americas would correspond with the separation and later diversification of the species belonging to the two groups of *Trypanosoma*. The few species of the *T. cruzi* clade present in the Old World would have been unique to bats and therefore, dispersed as a result of their host migrations. However, Hamilton et al. (2009) demonstrated infections in African terrestrial mammals by *Trypanosoma* species from the *T. cruzi* clade. This finding, associated with the fact that the vast majority of the species within this clade is exclusively described in bats and based on the phylogenetic inferences of the known *Trypanosoma* spp. in this clade, presented “The Bat Seeding Hypothesis” that proposed bats as the ancestral hosts of *T. cruzi* and *T. rangeli* (Hamilton et al. 2012).

Although not currently accepted as an explanation for the origin of the *Trypanosoma* genus, the southern supercontinent hypothesis is accepted to explain the origin of the *Trypanosoma* infection in marsupials, some of the most ancient hosts of these parasites. Actually, the ancestors of the currently known marsupial species originated in the portion of the supercontinent that would eventually become the Americas and probably dispersed to the areas that now comprise Antarctica and Oceania. It is expected that the parasites infecting these ancestral species likely dispersed with their hosts. Opossum species of the genus *Didelphis* (Didelphidae) would have originated after continental separation and are contemporary representatives of South America's native marsupial fauna (Mitchell et al. 2014). On this continent, and also in Central and North America, these opossums have a wide distribution, occupying distinct niches in natural and disturbed environments. Partially because of their synanthropic characteristics, they are some of the most studied hosts with regard to infections with species of *Trypanosoma* and *Leishmania*.

In a pioneering study, Noyes et al. (1999) found a putatively new trypanosome species (H25) that infected the eastern grey kangaroo (*Macropus giganteus*) in Australia, which was recently described as *T. noyesi* (Botero et al. 2016). Since then, molecular tools have been widely employed to describe new *Trypanosoma* species, using blood as the main source of DNA. Some new species have been described in recent years, especially in Australia, but the majority have been identified based only on their molecular characteristics, without parasite isolation. This leaves some important biological aspects of these parasite species unknown. Only in a few instances new species of *Trypanosoma* have been isolated and morphologically analysed (Noyes et al. 1999, McInnes et al. 2011, Lima et al. 2013, 2015).

In this study, we used an integrative approach to assess a new trypanosome species that was obtained from the spleen and liver tissues of a *Didelphis aurita* captured at Campus Fiocruz Mata Atlântica (CFMA), Rio de Janeiro, Brazil. This included the isolation of the parasite, as well as its characterisation and DNA sequencing. Through phylogenetic analyses, *Trypanosoma janseni* n. sp. provides new data on the evolutionary history of the origin of the *T. cruzi* clade.

## MATERIALS AND METHODS

*Parasites* - Two populations of flagellated parasites (17665B and 17665F) were isolated from the spleen and liver fragments of *D. aurita* (common opossum), during a field expedition conducted at Campus Fiocruz of Mata Atlântica (Rio de Janeiro, Brazil) in July 2012. This specimen was an adult male (with complete dentition and 1.4 kg of body weight), captured using a Tomahawk® live-trap (Tomahawk Live Traps, Tomahawk, WI, USA) in a transition area between peridomiciliary and forest areas (22°56′27.89″S; 43°24′23.17″W). Both parasite populations were isolated using biphasic media composed of NNN (Neal, Novy, Nicolle) and Schneider's media with 10% foetal bovine serum (FBS), maintained in a BOD chamber at 28°C.

*Culture medium selection and parasite growth curve* - Five distinct types of culture media were supplemented with 10% FBS and tested to establish *in vitro* cultures of the parasites: DMEM (Dulbecco's Modified Eagle's Medium), EAGLE (minimum Eagle Medium), TRPMI (Roswell Park Memorial Institute, commercial cell culture medium supplemented with tryptose), RPMI 1640 (Roswell Park Memorial Institute, commercial cell culture medium), and LIT (Liver Infusion Tryptose) (Noyes et al. 1999). We also tested the feeder-layer technique (employed for distinct, non-cultivable trypanosome species), in which monolayers of Vero cells and RPMI 1640 media were used with 10% FBS (v/v).

A growth assay was performed in triplicate, in two distinct biological replicates, adapting the methodology described in Araújo et al. (2007) using Schneider's medium complemented with 10% (v/v) of FBS and 2% (v/v) human male urine. The parasites were grown in test tubes containing 5 mL of the aforementioned culture media inoculated with 1 × 10^6^ parasites. The parasites were incubated at 27°C and counted daily using a Neubauer haemocytometer, until the day after the doubling time. Culture smears at the exponential phase of *in vitro* growth (day 2) were made in triplicate, Giemsa-stained, and observed under a Zeiss AxioObserver M1 microscope (Oberkochen, Germany).

*Ultrastructure analysis* - For transmission electron microscopy (TEM) analyses, isolates 17665B and 17665F were grown to exponential phase (5 × 10^7^ cells), washed thrice with PBS, and fixed in 2.5% glutaraldehyde (GA) in 0.1 M sodium cacodylate buffer (pH 7.2) for 40 min at 25°C. Post-fixation, cells were treated with 1% osmium tetroxide (OsO_4_) in 0.1 M sodium cacodylate buffer (pH 7.2) containing 0.8% potassium ferricyanide and 2.5 mM calcium chloride for 20 min at 25°C. Samples were then dehydrated through a graded acetone series (50%, 70%, 90%, and twice at 100%), and embedded in Poly/Bed 812 resin. Ultrathin sections were contrasted with 5% uranyl acetate (20 min) and lead citrate (2 min) prior to examination with a JEOL JEM-1011 transmission electron microscope (Tokyo, Japan). Alternatively, for scanning electron microscopy (SEM) analysis, fixed parasites were adhered to poly-L-lysine-coated coverslips, post-fixed with 1% OsO_4_, and dehydrated through a graded ethanol series (50%, 70%, 90%, and twice at 100%).

The samples were dried using a critical point method with CO_2_, mounted on aluminium stubs, coated with a 20 nm-thick gold layer, and examined with a JEOL JSM-6390LV scanning electron microscope (Tokyo, Japan). Ultrastructure analyses were performed at Plataforma de Microscopia Eletrônica, IOC, FIOCRUZ. Morphometric data were taken from 50 SEM images obtained from each isolate using the *Image J* program v. 1.47. The measured parameters were the size of the body and flagellum and the total size of the parasite.

*Nested polymerase chain reaction (PCR) targeting 18S SSU, PCR targeting glycosomal glyceraldehyde-3-phosphate dehydrogenase (gGAPDH), and DNA sequencing of both PCR products* - Nested PCR targeting a portion of the variable region of the small subunit ribosomal gene (18S SSU) was performed in two rounds. For the first round, each 50 µL reaction contained 2 µL of DNA (50 ng/µL), 10 µL of 5x polymerisation buffer with 100 mM dNTPs, 3 mM MgCl_2_, 1.4 U of *Taq* DNA polymerase (Promega, Madison, USA), and 16 *p*-mol of the following external primers: TRY927F (5′-GAAACAAGAAACACGGGAG-3′) and TRY927R (5′-CTACTGGGCAGCTTGGA-3′). Thermal cycling was conducted in an Esco Swift MaxPro Thermal Cycler SWT-MXP+SWY-MXP-BL-C for 30 cycles at 94°C for 30 s, 55°C for 60 s, and 72°C for 90 s. Products from the first amplification were diluted (1:10) in sterile deionised water. For the second round of PCR, 2 µL of this dilution was used as a template with the following internal primers: SSU561F (5′-TGGGATAACAAAGGAGCA-3′) and SSU561R (5′-CTGAGACTGTAACCTCAAAGC-3′), using the same PCR reaction mixture and cycle conditions described above (Smith et al. 2008). Amplified products were separated by molecular weight on a 1.5% agarose gel run at 100 V for 1 h in Tris-acetate EDTA buffer, stained with ethidium bromide, and visualised by illumination with UV light. Samples producing a band of approximately 600 bp were considered positive. For the gGAPDH target, we performed amplification on an 800 bp portion of the gene as previously described (Borghesan et al. 2013). Reaction mixtures (50 µL) contained ~100 ng of DNA, 100 ng of each primer, 200 M of each dNTP, 1.5 mM MgCl_2_, and 2.5 U of *Taq* DNA polymerase. Reactions were run for 30 cycles of 1 min at 94°C, 2 min at 48°C and 2 min at 72°C, with an initial cycle of 3 min at 94°C and a final cycle of 10 min at 72°C in the aforementioned thermocycler.

Amplified products were separated by molecular weight on a 1.5% agarose gel at 100 V for 1 h in Tris-acetate EDTA buffer and stained with ethidium bromide. Bands were visualised on an automated gel imaging system, Gel Logic 212 Pro, using the Carestream MI SE image software (Carestream, Rochester, NY, USA). Samples with single, clear bands at approximately 600 bp and 800 bp for 18S SSU and gGAPDH, respectively, were considered positive.

Both molecular targets were purified using the Wizard^®^ SV Gel and PCR Clean-Up System (Promega, Madison, USA) following the manufacturer's instructions. Both strands were subjected to Sanger sequencing reactions (ABI PRISM^®^ BigDye^®^ Terminator v.3.1 Cycle Sequencing Kit, Applied Biosystems) and run on an ABI 3730 sequencer (Applied Biosystems^®^, California, USA) at the DNA Sequencing Platform PDTIS/Fiocruz.

*Phylogenetic and distance analyses of SSU rRNA and gGAPDH genes* - For each gene, we visually inspected the forward and reverse DNA strands and generated consensus sequences using SeqMan Lasergene v.7.0 (DNASTAR Inc., Madison, Wisconsin, USA). These sequences were compared against the NCBI database (https://blast.ncbi.nlm.nih.gov) with the BLASTn algorithm, using values > 94% and > 96% as the identity and coverage cut-offs, respectively. We aligned our sequences with the sequences retrieved from the GenBank database in MAFFT v.7.0, using the L-INS-i algorithm (Katoh & Standley 2013). The 18S SSU alignment was visually inspected and manually edited to improve accuracy, especially in highly variable regions. Due to the high variability and low-confidence homology of a 165 bp fragment from the total 737 bp 18S SSU alignment, this region was excluded from the analysis (Supplementary data, Fig. 1).

We assessed the level of base substitution saturation in gGAPDH and 18S SSU sequences by calculating the entropy-based index implemented in DAMBE v.6. In brief, an index of substitution saturation (*I_SS_*) is calculated using the alignment data and compared to a critical index of substitution saturation (I_SS.C_), which determines a threshold for significant saturation of the data. If *I_SS_* is not significantly lower than *I_SS.C_*, sequences have experienced severe substitution saturation.

Possible recombinant regions in gGAPDH and 18S SSU sequences were identified with the Recombination Analysis Tool (Etherington et al. 2005) that uses a distance-based method of recombination detection, in which crossover points can be visually inspected in the distance plots. We used different species that belong to the *T. cruzi* clade as query for comparisons. All crossover points were statistically tested with the Genetic Algorithm for Recombination detection (Pond et al. 2006) through the assessment of the goodness of fit with the Akaike information criterion (AIC), derived from a maximum likelihood model fit to each segment.

Akaike and Bayesian information criteria of the jModelTest v.2 were used to choose Tamura-Nei (Tamura & Nei 1993) with four gamma categories (TrN+Γ) with unequal nucleotide frequencies and TrN+Γ with equal frequencies of nucleotides as the best-fit evolutionary models for the gGAPDH and 18S SSU datasets, respectively.

We tested whether a strict or a relaxed molecular clock best fit our data through a Bayesian random local clock analysis (RLC) (Drummond & Suchard 2010) with BEAST v.1.8. Three independent runs were performed for 2 × 10^9^ generations, sampling every 20,000 generations. Convergence of parameters and proper mixing were confirmed by calculating the effective sample size (ESS) in TRACER v.1.6, excluding the initial 10% (burn-in) of each run. All considered parameters had ESS > 500.

Phylogenetic trees were reconstructed with both maximum likelihood (ML) and Bayesian methods. We analysed each gene separately and also gGAPDH + 18S concatenated with SeaView (Gouy et al. 2010). For ML reconstructions in PHYML v.3.0, we used both the Nearest Neighbour Interchange and the Tree Bisection and Reconnection algorithms to improve tree searching. Branch supports were assessed via 1000 bootstrap replicates.

We used a Bayesian Markov chain Monte Carlo (MCMC) method for species coalescent analysis based on multilocus data (18S SSU and gGAPDH). We inferred both 18S SSU and gGAPDH gene trees, concatenated sequences tree, and also a species tree with *BEAST (Heled & Drummond 2010), which is included in the BEAST v.1.8 package. Information in the literature about the taxonomy of DNA sequences retrieved from GenBank was used to assign prior information on each sequence to a species. In the case of sequences without taxonomic identification at the species level, we maintained each sequence as a single “fictitious species” (Supplementary data, Table). We used *Herpetomonas muscarum* sequences to root phylograms.

The Yule-coalescent model of speciation was imposed in tree reconstructions to assign individuals to species. This is the simplest birth-death model of speciation, which considers each tree node as a speciation process. Therefore, it is the best choice for phylogenies with many species represented by a few sequences in a species tree based on one or few molecular markers (Ogilvie et al. 2016). Number of independent runs, parameter sampling, and inspection of parameter convergence were identical to RLC analysis. Runs were combined using LogCombiner and a maximum clade credibility (MCC) tree based on 10,000 trees (burn-in = 2,000) was generated for both gene fragments with a posterior probability limit of 0.6 using Tree Annotator (both part of the BEAST package). Statistical support for clades was assessed by the posterior probability (PP) method. All resulting species trees were visualised in Figtree v.1.4 (http://tree.bio.ed.ac.uk/software/figtree/).

Species distance matrices were estimated for both 18S SSU and gGAPDH fragments using the Tamura-Nei substitution model, available in MEGA 5. Distance variances were estimated with 1,000 bootstrap pseudo-replicates.

The parameters that we believed represented valid species included sequences that (i) clustered into a single and well-supported monophyletic clade (*i.e.*, bootstrap ≥ 60% and posterior probability ≥ 0.8), and (ii) were genetically more distant from another species instead of the minimal genetic distance observed for two *bona fide Trypanosoma* species.

*Estimation of divergence times* - We tested single and multiple calibration points based on previous phylogenetic data (Stevens & Rambaut 2001, Lewis et al. 2011) to infer divergence times. Log marginal likelihood Bayes Factor (LBF) was used to compare the marginal likelihood of each hypothesis, estimated with the path sampling and stepping stone algorithms (Baele et al. 2013). We also sampled from all prior hypotheses (*i.e.*, analysis with only priors and no sequence data) to make sure that all priors were proper and together, they did not produce an unexpected joint prior. Moreover, these results were used to estimate the influence of the prior and the sequence data on posterior results.

*Ethics* - The mammal capture was licensed by the Brazilian Institute of Environment and Renewable Natural Resources (IBAMA/CGFAU/LIC; license 3665-1). Tissue sample collection and euthanasia were performed as regulated by the Federal Counsel of Medical Veterinary under resolution number 1.000 approved on May 11th, 2012, and the procedures were approved by the FIOCRUZ Committees of Bioethics (LW81/12).

## RESULTS

**Fig. 1:**
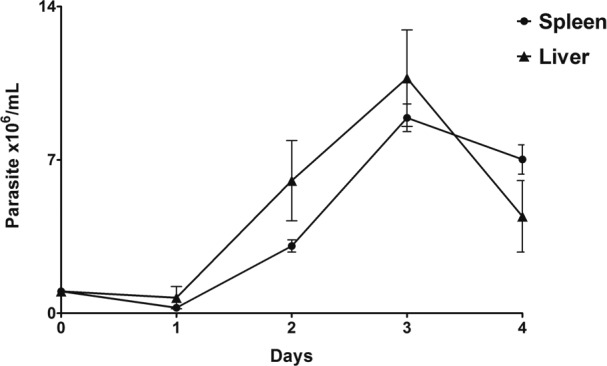
*in vitro* growth curve of *Trypanosoma janseni* epimastigotes derived from the spleen and liver of *Didelphis aurita*. Growth assay was performed in Schneider's medium containing 2% human male urine and 10% of FBS and presented a log phase from day 1 to 3.

*Parasite viability, growth behaviour, and morphology* - The *in vitro* parasite growth assay generated a very short log phase and an absent stationary phase. As shown in Fig. 1, parasite populations from the spleen and liver remained unchanged in the first 24 h, followed by a rapid increase in parasite populations on day 2. Both parasites (isolates 17665B and 17665F) achieved a maximum growth rate at day 3, followed by a sharp decline. Based on these results, we established that parasites should be passaged on the third day of culture. The growth curve was performed only until day 4 because the parasite population started to decrease and present more degenerative forms than viable parasites after that. In an attempt to find a nutrient medium which would promote better growth of the parasites *in vitro*, we tested five different nutrient media (DMEM, EAGLE, TRPMI, RPMI 1640, and LIT) supplemented with 10% FBS, as well as a Vero cell monolayer culture. Neither the tested culture media nor the Vero cell culture succeeded in the long-term maintenance of the parasites in culture. The maximum length of time in which motile parasites were observed was 48 h, although most of the parasites were already dead after the first 24 h. On the third day, no living cell was observed in any of the tested media.

**Fig. 2:**
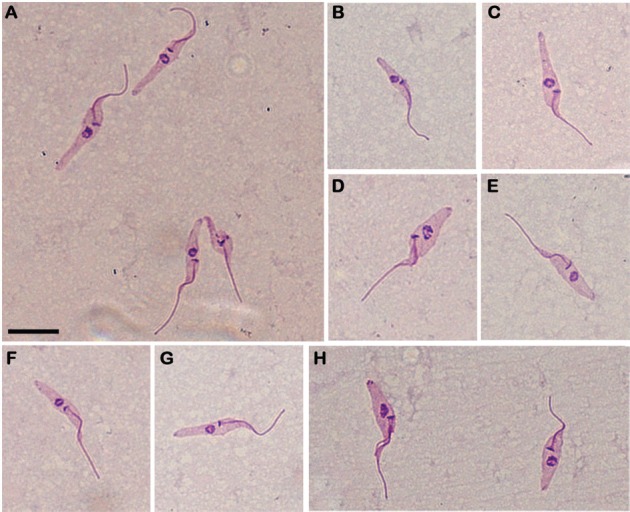
axenic culture epimastigotes of *Trypanosoma janseni* n. sp. derived from the spleen tissues of *Didelphis aurita* (a-h). Smears were performed with parasites in the logarithmic phase of the growth curve and Giemsa-stained. Scale-bar =10 µm.

Another approach was the morphological analysis of these parasites, which was performed on day 2 of the growth assay (exponential phase). In Giemsa-stained culture smears, only epimastigotes could be observed (Fig. 2). The cells had the characteristic kinetoplast localised in the anterior portion of the parasite, near the nucleus. The established culture was fairly homogeneous in form, but not in size, showing large variation in body size.

**Fig. 3:**
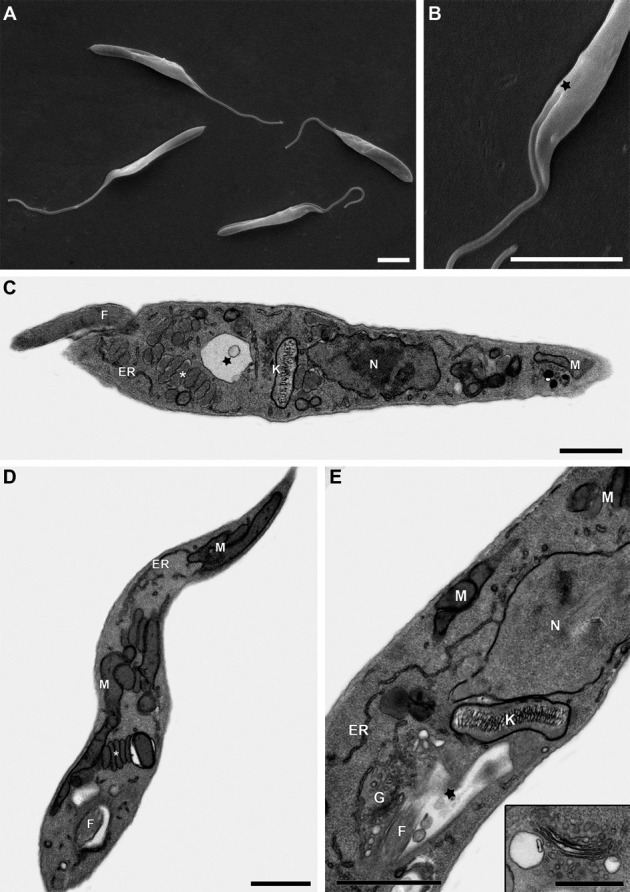
ultrastructure images of *Trypanosoma janseni* n. sp. epimastigotes. Scanning electron microscopy micrographs revealed elongated cells with slight body rotation (a) and a flagellar pocket on the anterior position (a-b), characteristic of epimastigote forms (b; black star). Transmission electron microscopy micrographs showing unique and branched mitochondria (c, d, and e), an observed stem-shaped kinetoplast, as well as a nucleus, endoplasmic reticulum, Golgi apparatus, flagellum, and flagellar pocket. Saccular or elongated structures presented in numerous stacks (white asterisk) can also be observed. In detail, Golgi cisternae (e; inset). Abbreviations: M = mitochondria, N = nucleus, K = kinetoplast, ER = endoplasmic reticulum, G = Golgi apparatus. Scale-bars in a-b = 5 µm; c-e = 1 µm; e (inset) = 0.5 µm.

*Ultrastructure and morphometric analysis* - Morphometric analysis using SEM images of the isolated parasites revealed no significant differences (p > 0.05) between the measured values of the two populations, 17665B and 17665F. For the two populations, the total length of the parasites was reported as [mean ± standard deviation (SD)] 23.3 ± 5.9 µm and 28.7 ± 6.2 µm, respectively, the length of the free portion of the flagellum was 9.5 ± 3.2 µm and 11.6 ± 3.0 µm, respectively, and the body length was 13.8 ± 3.6 µm and 17.1 ± 3.7 µm, respectively. The high SD values demonstrate the differences observed in parasite size among distinct cells in the same population. The morphology of all the cells was compatible with the epimastigote form of the parasite, showing the emergence of a lateral flagellum (Fig. 3A-B).

Cells from both cultures (spleen and liver) presented an elongated body with the nucleus in the anterior portion and a single flagellum as revealed by TEM (Fig. 3C). The kinetoplast shows similar stem morphology (Fig. 3C-D) in a well-defined region of the mitochondria, near the flagellar pocket, compatible with the epimastigote form of the parasite. Mitochondria are branched, appearing throughout the cell body with double membranes and mitochondrial cristae (Fig. 3C-E). The structure of the Golgi is similar to that found in eukaryotic cells (Fig. 3E, inset), with a set of stacked cisternae located close to the kinetoplast. The point of emergence of the flagellum was also observed. Moreover, an elongated, cellular, electron-dense structure was observed, with a simple membrane either present in small amounts or widely distributed throughout the parasite body, usually in sets of three or four (Fig. 3C-D).

*Molecular characterisation and phylogenetic inferences* - DNA extracted from both parasite populations were PCR-amplified using 18S SSU and gGAPDH targets. The DNA sequences obtained from the parasite populations derived from the spleen and liver were identical for the two targets, indicating that both isolates belong to the same species/genotype. BLASTn results using these sequences showed high similarity with *T. wauwau* (similarity > 95%; coverage > 99%), a species recently described in Neotropical bats (GenBank: KT030800, KT030801, KT030810, and KT030821). The sequences also showed similarity to other *Trypanosoma* species detected in marsupials of the families Potoroidae and Macropodidae from the West and Southeast parts of Australia (GenBank: JN315381, JN315382, JN315395, JN315396, AJ009168, and AJ620276 for *T. noyesi*, and KC753537, KC812988, KU354263, and KU354264 for *Trypanosoma* sp. sequences).

**Fig. 4:**
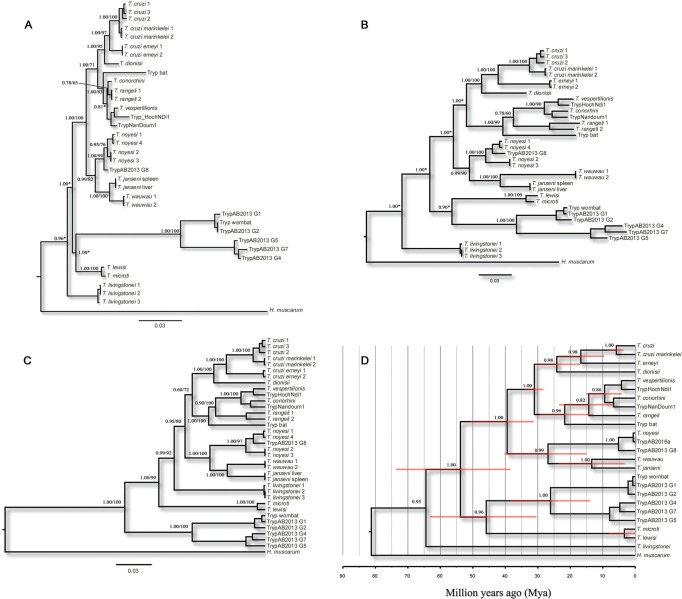
phylogenetic reconstructions based on Bayesian inferences (BI) and Maximum Likelihood (ML) methods. Phylogenetic trees based on the alignments of a 572 bp fragment of 18S SSU (A), gGAPDH (708 bp) (B), concatenated 18S + gGAPDH (1,280 bp) (C), and the maximum clade credibility timetree of the trypanosomatid species obtained from BEAST (D). Scale-bars (in a-c) indicate the number of substitutions per site. Red rectangles (in d) indicate 95% of the highest posterior distributions (HPD) for the time of splits. Posterior probabilities, followed by bootstrap values, are shown near the nodes (with the exception of d, where only posterior probabilities are shown). Posterior probabilities with an asterisk (*) indicate that ML topology was incongruent (see text for further details). Posterior probability, bootstrap values, and 95% HPD on tip nodes were omitted to better visualise the trees. GenBank accession numbers for each terminal taxon can be found in Supplementary data, Table.

In addition to those genetically similar sequences, we added sequences for other Trypanosomatidae into the phylogenetic analysis to obtain a better picture of the evolutionary position of our isolates [Fig. 4, Supplementary data (Table)].

**TABLE:** Calibration hypotheses and model probability results

Calibrations	Divergence time^*a*^ (Mean ± SD)	Marginal likelihood^*b*^	Model probability (LBF)
Diversification of *Trypanosoma cruzi* and *T. cruzi marinkelei*	6.5 ± 1.4 Mya	-	-
Diversification of *T. cruzi* and *T. rangeli*	31 ± 1.6 Mya	-7892.4349	47.8%
Diversification of *T. cruzi* and *T. rangeli*, and *T. cruzi* and *T. cruzi marinkelei*	31 ± 1.6 Mya and 6.5 ± 1.4 Mya	-7892.3465	52.2%

a normal distribution was assumed for all calibrations. Divergence times were chosen based on independent results of Stevens & Rambaut (2001) and Lewis et al. (2011);

b model probability was calculated with Log marginal likelihood Bayes Factor (LBF) to compare the marginal likelihood of each hypothesis. Results of marginal likelihood are the mean of three independent runs and were estimated with the path sampling and stepping stone algorithms (Baele et al. 2013);

SD: standard deviation.

Nonsignificant substitution saturation was found in both 18S and gGAPDH sequences (*I_SS_* = 0.06, *I_SS.C_* = 0.70, p < 0.0001, for 18S sequences; and *I_SS_* = 0.17, *I_SS.C_* = 0.72, p < 0.0001, for gGAPDH sequences). No sign of recombination was found in the 18S sequences, but distance plots demonstrated at least three crossover points in the gGAPDH sequences of the Australian *Trypanosoma* sp. specimens (strains AB2013 G4, G5, and G7) and *T. microti*, which might indicate possible recombination hotspots (Supplementary data, Fig. 2). The first crossover point at position 160 of the gGAPDH alignment was statistically significant (p < 0.001).

Phylogenetic reconstructions corroborated what was previously observed in recombination analysis. Indeed, preliminary analyses with ML and BI, assuming a strict clock, displayed incongruent topologies for 18S and gGAPDH sequences. *Trypanosoma* (*Schizotrypanum*) and *T. rangeli* clades were monophyletic in a well-supported clade in the 18S trees (BP = 71%; PP = 1.00; Fig. 4A), but paraphyletic in the gGAPDH trees. The exclusion of three Australian *Trypanosoma* sp. gGAPDH sequences (GenBank: KC812985, KC812986, and KC812987) from the analysis produced an identical topology to that observed in the 18S tree, *i.e.*, showing the monophyly of *Schizotrypanum* and *T. rangeli* clades.

RLC analysis indicated that gGAPDH sequences evolve almost twice as fast as 18S sequences (2.04 × 10^-3^ and 1.09 × 10^-3^ substitutions/(site million years), respectively). Moreover, both datasets exhibited changes in mutation rates across samples (mean rate changes of 2.67 and 2.55 for gGAPDH and 18S sequences, respectively). Therefore, the implementation of a ‘relaxed' clock was more suitable to our 18S and gGAPDH datasets. Indeed, when a relaxed clock was assumed, gGAPDH and 18S topologies were perfectly congruent (Fig. 4B).

It is noteworthy that irrespective of the methods used, all phylogenetic analyses showed that the parasite species described herein belongs to the *T. cruzi* clade, grouping with *T. wauwau* in a well-supported clade (BP = 100%; PP = 1.00). Both species are exclusive and reciprocally monophyletic. This clade (*T. janseni* n. sp. + *T. wauwau*) is a sister group to the Australian trypanosomes, *T. noyesi* and *Trypanosoma* sp. (BP = 100%; PP = 1.00).

A multispecies coalescent model in *BEAST with a ‘relaxed' clock and ML tree with concatenated data was consistent with gene tree reconstructions assuming a ‘relaxed clock' and thus, provided a good fit to the 18S SSU and gGAPDH data (Fig. 4C-D). Our samples were clearly separated from *T. wauwau* (BP = 100%; PP = 1.00). Moreover, genetic distances between *T. janseni* and *T. wauwau*, based on the 18S SSU and gGAPDH sequences [(mean distance ± variance) 1.7 ± 0.5% for 18S SSU and 9.7 ± 1.2% for gGAPDH], were similar to those observed between *T. erneyi* and *T. dionisii* (2.4 ± 0.7% and 9.2 ± 1.2%, respectively), *T. erneyi* and *T. cruzi marinkellei* (1.8 ± 0.6% and 9.5 ± 1.3%, respectively), and *T. conorhini* + *T. rangeli* and *T. vespertilionis* (2.4 ± 0.6% and 7.0 ± 3.5%, respectively). Altogether, these results provide strong evidence that *T. janseni* represents a *bona fide* species within the *T. cruzi* clade.

*Estimated dates for the evolutionary history of the T. cruzi clade* - MCMC chains did not converge to a stationary distribution in important parameters of the tree (such as posterior, likelihood, and clock rate) when we used the single calibration point based on the split between *T. cruzi* and *T. cruzi marinkellei*, sampling more chains, optimising parameters from previous runs, or increasing the number of chains. Therefore, this model was excluded from the analysis.

Marginal likelihood values (and consequently, model probabilities calculated with LBF) were quite similar for the trees estimated with a single calibration point based on the split between *Trypanosoma* (*Schizotrypanum*) and *T. rangeli* clades. With respect to two calibration points, the same was observed based on the split between *Schizotrypanum* and *T. rangeli* clades and between *T. cruzi* and *T. cruzi marinkellei* (Table).

According to the outgroup comparison, the *T. cruzi* clade was estimated to have taken place at ~81 Mya (CI: 123-52 Mya; Fig. 4D). The separation of the South American *T. wauwau* + *T. janseni* from the Australian *T. noyesi* clade (*T. noyesi* + AB2016a + AB2013 G8) was dated at ~ 26 Mya (CI: 40-15 Mya).

Class Kinetoplastea Konigberg, 1963

Order Trypanosomatidae (Kent 1880) Holande, 1952

Family Trypanosomatidae Doflein, 1901

Genus *Trypanosoma* Gruby, 1843

*Trypanosoma janseni* n. sp. (Figs. 2-3)

*Description* - Only epimastigotes were observed in culture. The evolutive form was confirmed by the position of the flagellum output. *T. janseni* n. sp. cells showed a small and elongated appearance, with a slightly twisted body and a single flagellum, a nucleus in the posterior portion, and a kinetoplast showing similar stem morphology localised in the anterior portion of the parasite, near the nucleus. Mitochondria appear branched throughout the cell body with double membranes and mitochondrial cristae. The structure of the Golgi is similar to that found in eukaryotic cells, with a set of stacked cisternae located near the kinetoplast and the output of the flagellum. We also observed various endosomal vesicles similar to acidocalcisomes positioned near the flagellar pocket. Additionally, there was an elongated, cellular, electron-dense structure, with a simple membrane present either in small quantities or widely distributed throughout the protozoan body, usually in sets of three or four.

*Type host - Didelphis aurita* (Wied-Neuwied, 1826)

*Site in the host* - Spleen and liver

*Type locality* - Atlantic Rainforest biome, Rio de Janeiro/RJ, Brazil (22°56′27.89″S; 43°24′23.17″W).

*Type data and depository* - Hapantotype; cultures of parasite populations derived from the spleen and liver are deposited in the Coleção de Trypanosoma de Mamíferos Silvestres, Domésticos e Vetores, COLTRYP/FIOCRUZ (www.coltryp.fiocruz.br) under the accession numbers COLTRYP 715 and 716. The newly generated sequences were deposited in the GenBank database under the accession numbers KY243025 and KY243026 (18S rRNA gene) and KY549444 and KY549445 (gGAPDH gene).

*Vector* - Unknown

*ZooBank registration* - In accordance with section 8.5 of the International Code of Zoological Nomenclature (ICZN), details of the new species have been submitted to ZooBank with the life science identifier (LSID) zoobank.org:pub: 61AA3E83-7ECB-408B-8955-499EEBF4EAC4.

*Etymology* - The name *T. janseni* n. sp. was given in honour of Dr Ana Maria Jansen, a distinguished parasitologist from Brazil, who worked for several years with opossums as experimental models. Having an animal facility to study this mammalian host not only provided her with material for several manuscripts, but also 15 years of legal issues until she proved that everything was properly, legally, and ethically conducted. With this new species name, we recognise the merit of a researcher that persistently and painstakingly endeavoured to investigate all possible factors involved in the very complex trypanosome life-cycle.

## DISCUSSION

Small mammals have been described as important reservoirs of different trypanosomatids such as *T. cruzi*, *T. rangeli*, and *Leishmania* spp. In recent years, several studies have investigated the importance of these hosts in maintaining the sylvatic cycle of the *Leishmania* spp. The role of the *Didelphis* spp. as *Leishmania* reservoirs was already proposed. In studies with *Leishmania* spp., the gold standard for isolating parasites included the cultivation of hematopoietic tissues (such as the spleen, liver, and bone marrow) using Schneider's media with 10% FBS. On the other hand, *T. cruzi* is usually isolated from blood samples of the infected hosts. Aiming to diagnose and isolate both trypanosomatids, we routinely cultivate blood, liver, skin, and spleen samples from small mammals that we capture during field work. Here, we isolated parasite populations from the blood, spleen, and liver samples from a *D. aurita* captured in the Atlantic Rainforest of Rio de Janeiro, Brazil. This mammalian host displayed mixed infection, as it was infected with *T. cruzi* in the blood (as demonstrated by haemoculture; data not shown) and with the new species described here, in the spleen and liver. Its vector is unknown, but it is probably associated to marsupials because dixenous haemoflagellates usually adapt their transmission to ecological conditions and environment of their hosts. Alternatively, *T. janseni* may be a monoxenous trypanosomatid in the process of pre-adaptation to a mammal host (Lukeš et al. 2014).

It was very surprising that a new *Trypanosoma* species could be described in one of the most intensively studied reservoirs of *T. cruzi* and *Leishmania* spp. in the last century. One hypothesis is that the co-infection with *T. cruzi* may have altered the host immune responses, resulting in a population increase of *T. janseni* n. sp., allowing its isolation in axenic culture. Another possible reason for the detection of *T. janseni* n. sp. is the fact that we were searching for *Leishmania* parasites, which led us to use Schneider's medium complemented with 10% FBS as culture media. It is also possible that if the same tissues of this host were also infected with the *Leishmania* sp., the *in vitro* growth of the latter probably would have supplanted the population of *T. janseni*, hampering its detection. Furthermore, most studies aiming to identify *Trypanosoma* parasites investigate only blood samples, as observed in the Australian studies of trypanosomes (McInnes et al. 2011). Multiple variables can act as a biological filter by selecting the parasitic populations that could be isolated, both inside a host or under *in vitro* conditions. Our success in the isolation of this new species of *Trypanosoma* was the result of a combination of factors, and we are thus not able to note the most important one.

Another important point is that we know nothing yet about the biology of this new parasite in nature. We are also unsure if the epimastigote form observed in the culture was the unique form. However, a trypomastigote form is surely expected and we believe that *T. janseni* n. sp. also possesses this morphological form under particular conditions in the host and/or axenic culture that we were unable to mimic under laboratory conditions. We tried different culture media, but did not observe a transition from epimastigote to other morphological forms. In addition, we cannot infer anything about the parasite's course of infection in a host co-infected by another *Trypanosoma*, as was the case with *T. cruzi*. Even if *T. janseni* n. sp. has the ability to colonise other tissues, such as blood, the presence of *T. cruzi* could be influencing this colonisation. However, having been isolated in highly vascularised tissues, parasitic populations present in the spleen and liver could be present in the microvessels of those tissues. Spleen microvessels, for example, are a common site for *T. musculi* infections (Albright et al. 1999). In a preliminary experiment, we infected cultured peritoneal macrophages, but infection was not established (data not shown).

SEM images confirmed that the morphological characteristics are similar to axenic epimastigote forms. The morphometric analysis also showed an intense polymorphism, with widely ranging body length and width between cells in the same isolate. The cells showed a small and elongated appearance, with a slightly twisted body, similar to *T. livingstonei* (Lima et al. 2013). All *Trypanosoma* parasite stages present a single flagellum that emerges from the anterior region, kinetoplast, branched mitochondria, flagellar pocket, basal bodies, subpellicular microtubules, paraflagellar rod, and acidocalcisomes (de Souza 2008). The ultrastructure analysis showed most of these structures, along with an unidentified cell structure with no parallel in other trypanosomes, which presented as single membrane structures usually grouped in stacks of three or four. To the best of our knowledge, the structure we observed herein (although displaying differences in size and shape) resembles the glycosomes of the *Phytomonas* sp. isolated from *Euphorbia characias* (Attias & de Souza 1995). However, further studies must be carried out to characterise this structure.

Another component of the integrated approach employed here was the molecular taxonomy based on a partial region (737-bp) of the small subunit (18S) ribosomal RNA gene and 708 bp of the nuclear marker glycosomal glyceraldehyde-3-phosphate dehydrogenase (gGAPDH) genes. These targets are the most suitable choice to establish the taxonomic portion of a given trypanosomatid flagellate (Lukeš et al. 2014). Phylogenetic analyses of both markers with a ‘relaxed' clock and concatenated data showed similar topology, with small discrepancies. In general, gene, concatenated trees, and species trees showed the following: (i) the populations of the spleen and liver parasites belong to the same species; (ii) this species clusters with *T. wauwau*, a species recently described in Neotropical bats (Lima et al. 2015), in a well-supported clade where they are exclusive and monophyletic; and (iii) the genetic divergence between *T. wauwau* and *T. janseni* is similar to those observed for interspecific comparisons among trypanosomes. Cumulatively, these findings clearly demonstrate that these isolates represent a new *Trypanosoma* species (Votýpka et al. 2015).

Phylogenetic ML and Bayesian trees based on concatenated data showed *T. livingstonei* closer to the rest of the *T. cruzi* clade, followed by *T. lewisi* and *T. microti*, and Australian trypanosomes (AB2013 G1, G2, G3, G4, G5, and G7, and *Trypanosoma* sp. wombat) as a basal clade, such as previously observed by Lima et al. (2015). Gene and species trees with a ‘relaxed clock' clustered *T. lewisi* + *T. microti* and Australian trypanosomes together in a clade, and *T. livingstonei* sequences were positioned in a basal clade. Our results did not fully elucidate if this discrepancy was due to the recombination in gGAPDH sequences of these specimens, or if the rapid evolution of these lineages possibly distorted the topology of the tree, by long-branch attraction (Noyes & Rambaut 1998).

Most clonal species undergo recombination (Omilian et al. 2006), but it seems to be infrequent in the trypanosomatid taxa (Weir et al. 2016). We observed one crossover point at position 160 of the gGAPDH sequences in the Australian *Trypanosoma* sp. specimens (strains AB2013 G1, G2, G4, G5, and G7) and *T. microti*, which promotes significant topological incongruence (p < 0.001). However, excluding this and neighbour sites, *Trypanosoma* (*Schizotrypanum*) and *T. rangeli* groups remained paraphyletic, *T. lewisi* + *T. microti* and Australian trypanosomes were clustered in a monophyletic clade, and *T. livingstonei* sequences were maintained in a basal clade. Only the exclusion of the Australian *Trypanosoma* spp. gGAPDH sequences mentioned above or the application of a ‘relaxed' clock grouped *T.* (*Schizotrypanum*) and *T. rangeli* as a monophyletic clade. Resolving the correct phylogenetic position of these Australian samples, *T. livingstonei*, *T. microti*, and *T. lewisi* will require additional sampling, inclusion of more outgroups, and analysis of more nuclear markers.

Mutation rate estimations under the ‘relaxed' clock assumption are very close to previous estimations of 18S and other nuclear trypanosomatid sequences (Stevens & Rambaut 2001, Lewis et al. 2011, Weir et al. 2016). Assuming a ‘relaxed' clock model, it was possible to observe that, in general, gGAPDH evolves twice as fast as 18S.

Fossils and geological data suggest that marsupials have originated in South America and dispersed, together with their parasites, several times between the Americas and Australia during the period when both formed a southern supercontinent along with Antarctica (McInnes et al. 2011). Some authors also believe that marsupials play an important role in the diversification of trypanosomatids because they are natural hosts for dixenous parasites. Some monoxenous species could also preadapt to the dixenous life cycle as they can survive and multiply in the marsupial anal scent glands, which protects them from the host immune system and provides an ideal lower body temperature environment (Lukeš et al. 2014). Our phylogenetic analysis dated the separation of the Australian *T. noyesi* and South American *T. janseni* n. sp. and *T. wauwau* at ~26 Mya (CI: 40-15 Mya). The upper limit calculated here coincides with the opening of the South Tasman Rise and Drake Passage, which led to the complete separation of Australia, Antarctica, and South America around 45-40 Mya. This result would possibly explain why some Australian trypanosomes present high similarity with *T. janseni* n. sp., a trypanosome isolated from a South American marsupial.

The presence of *T. janseni* n. sp. in the same clade as the recently described bat trypanosome, *T. wauwau*, re-opens the question of the origin of the *Trypanosoma* species from indigenous Australian mammals. Instead of having evolved from a bat trypanosome, as proposed by Lima et al. (2015), those Australian marsupial *Trypanosoma* species might have originated from South American trypanosomes, as suggested by the similarity between *T. janseni* and some of the Australian trypanosomes. In this proposed scenario, South American marsupials infected with the ancestral lineage of *T. janseni* and *T. wauwau* would have entered Australia, and the parasite would have dispersed among Australian marsupials. Later, diversification gave rise to *T. janseni* and the closely related *Trypanosoma* species in Australian marsupials, which further gave rise to *T. noyesi*. *T. janseni* would have been maintained in marsupial species from the southern supercontinent, and a spill-over event to bats from a common ancestral lineage would have formed *T. wauwau* and the other relatives of the bat *Trypanosoma* species. In this scenario, *T. janseni*, along with *T. wauwau*, are the “missing-links” that shed light on the initial diversification process based on what we currently known about the *T. cruzi* clade.

The history of the evolution of trypanosomes has been changing, in tandem with the emergence of molecular tools with more discriminatory power. The total *Trypanosoma* universe from the *T. cruzi* clade is immense, subsampled, and therefore, still unknown. The more effort that is put into the search for trypanosomatids, more pieces of this puzzle will be revealed. Considering the information obtained, the hypothesis proposed here appears to be a more parsimonious explanation for some of the most ancestral species within the *T. cruzi* clade.

*In conclusion* - The description of a new parasite species must include, at least, morphological and molecular data to follow the “best practices” of the International Code of Zoological Nomenclature. In this work, we integrate classical taxonomy and phylogenetic tools to characterise a new species of parasite, *T. janseni*. This species was isolated from the spleen and liver tissues of the opossum *D. aurita* in the Atlantic Coastal Rainforest of the Rio de Janeiro municipality in Brazil. The parasite populations were isolated and maintained in Schneider's medium complemented with 10% (v/v) FBS and 2% (v/v) human male urine. It was determined that the parasite should be passaged on the third day of *in vitro* culture. Only epimastigote forms were observed, and the ultrastructure analysis showed most of the structures described in the other species within the *Trypanosoma* genus. Additionally, an unknown feature, which presented as single membrane structures usually grouped in stacks of three or four and very similar to the glycosomes of the *Phytomonas* sp. isolated from *Euphorbia characias*, might be a diagnostic characteristic for *T. janseni*. Phylogenetic analyses of the partial region of the lower ribosomal subunit 18S gene and gGAPDH confirmed *T. janseni* as a new species within the *T. cruzi* clade and showed that this species clusters with *T. wauwau* in a well-supported clade, with a close relationship to trypanosomes isolated from Australian marsupials.
