# Supplementary material for: Trypanosoma janseni n. sp. (Trypanosomatida: Trypanosomatidae) isolated from Didelphis aurita (Mammalia: Didelphidae) in the Atlantic Rainforest of Rio de Janeiro, Brazil: integrative taxonomy and phylogeography within the Trypanosoma cruzi clade
Source: Mem Inst Oswaldo Cruz. 2018 Jan;113(1):45–55. doi: 10.1590/0074-02760170297 (PMC5719541; doi:10.1590/0074-02760170297)
Supplement: Supplementary file 1 [file 0074-0276-mioc-113-01-0045-Suppl01.pdf]

TABLE

18S SSU and gGAPDH sequences used in this study

Species	Codes	gGAPDH Accession numbers	18S SSU Accession numbers	References
Trypanosoma cruzi	*T. cruzi* 1, *T. cruzi* 2, *T. cruzi* 3	GQ140356, KT305796, KT305797,	AJ009149, CP015675, KT829462	Stevens et al. (1998), Cavazzana Jr et al. (2010), Franzen et al. (2012), Lima et al. (2015), Pinto et al. (2015)
T. cruzi marinkelei	*T. cruzi marinkelei* 1, *T. cruzi marinkelei* 2	GQ140360, KF557743	KR653213, KR653216	Cavazzana Jr et al. (2010), da Costa et al. (2016)
T. dionisii	*T. dionisii*	FJ649494	FN599058	McInnes et al. (2009), Hamilton et al. (2012)
T. erneyi	*T. erneyi* 1, *T. erneyi* 2	JN040968, JN040969	JN040989, JN040990	Lima et al. (2012)
T. conorhini	*T. conorhini*	AJ620267	AJ012411	Stevens et al. (1999), Hamilton et al. (2004)
T. vespertilionis	*T. vespertilionis*	AJ620283	AJ009166	Stevens et al. (1998), Hamilton et al. (2004)
T. rangeli	*T. rangeli* 1, *T. rangeli* 2	GQ140364, JN040973	EF071580, KT368799	da Silva et al. (2007), Cavazzana Jr et al. (2010), Lima et al. (2012, 2015)
T. noyesi	*T. noyesi* 1, *T. noyesi* 2, *T. noyesi* 3, *T. noyesi* 4	AJ620276, JN315395, JN315396, KU354264	JN315381, JN315383, KU354263, KX008320	Hamilton et al. (2004), Paparini et al. (2011), Botero et al. (2016)
T. janseni	*T. janseni* liver, *T. janseni* spleen	KY549444, KY549445	KY243025, KY243026	**This study**
T. wauwau	*T. wauwau* 1, *T. wauwau* 2	KT030800, KT030801	KT030848, KT030849	Lima et al. (2015)
T. lewisi	*T. lewisi*	AJ620272	AJ009156	Stevens et al. (1998), Hamilton et al. (2004)
T. livingstonei	*T. livingstonei* 1, *T. livingstonei* 2, *T. livingstonei* 3	KF192958, KF192960, KF192965,	KF192980, KF192981, KF192987	Lima et al. (2013)
T. microti	*T. microti*	AJ620273	AJ009158	Stevens et al. (1999), Hamilton et al. (2004)
Herpetomonas muscarum	*H. muscarum*	DQ092548	JQ359731	Yurchenko et al. (2006), Borghesan et al. (2013)
Trypanosoma sp.	TrypAB2013 G1, TrypAB2013 G2, TrypAB2013 G4, TrypAB2013 G5, TrypAB2013 G7, TrypAB2013 G8, Tryp wombat, Tryp bat, TrypNanDoum1, Tryp_HochNDi1	AJ620277, FM164793, FM164794, GQ140365, KC812982, KC812983, KC812985, KC812986, KC812987, KC812988	AJ012418, AJ620558, FM202492, FM202493, KC753530, KC753531, KC753532, KC753534, KC753536, KC753537	Stevens et al. (1999), Hamilton et al. (2005, 2009), Cavazzana Jr et al. (2010), Botero et al. (2013)

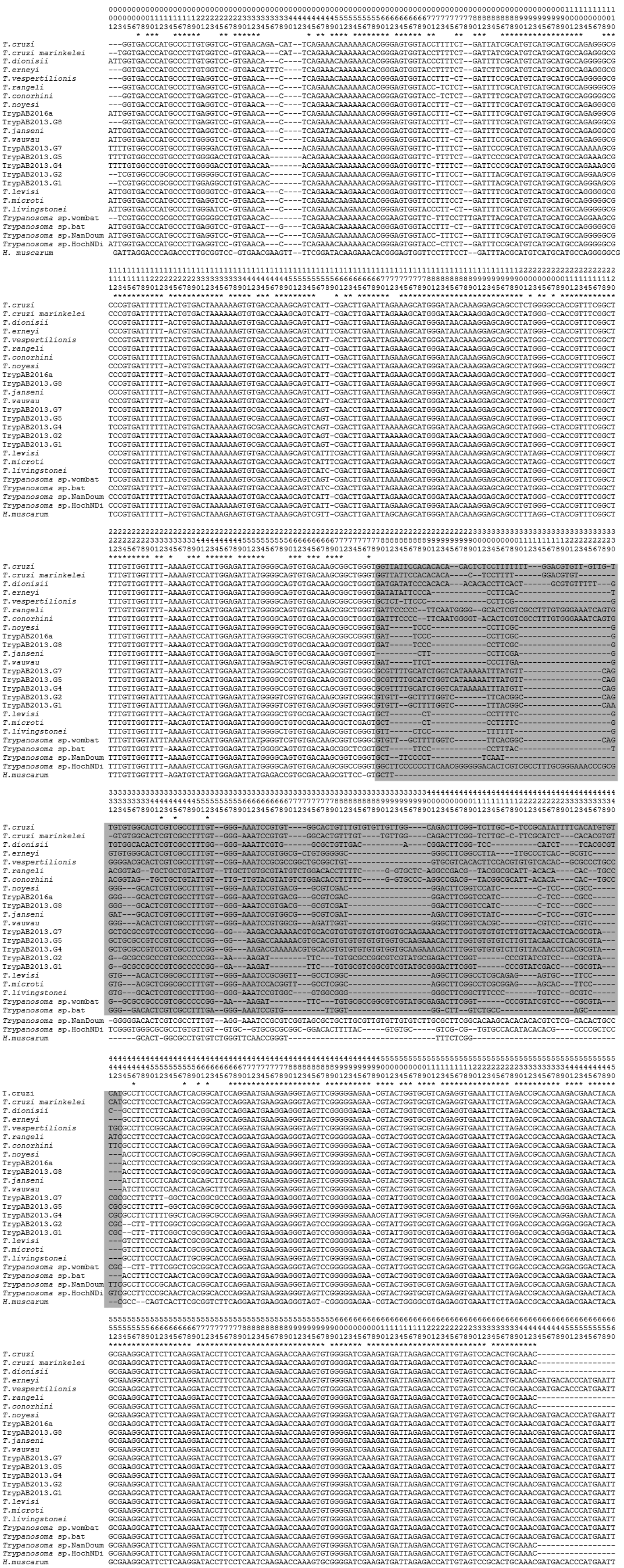

Supplementary data

```
                         66666666666666666666666666666666666666677777777777777777777777777777777777
                         66666666677777777778888888888999999999900000000001111111111222222222233333
                         12345678901234567890123456789012345678901234567890123456789012345678901234

T.cruzi                  --------------------------------------------------------------------------
T.cruzi marinkelei       --------------------------------------------------------------------------
T.dionisii               --------------------------------------------------------------------------
T.erneyi                 GGGGAGTTTTGGTCGC-AGGCGTGGTCGGGTT----CATCCCGTTCCTC-GTCTCGCCAATGAATATCAATTT
T.vespertilionis         GGGGAGTTTTGGTCGTTAGGCGAGGTCGGGTT----CATCTCGCTCCTC-GTCTCGCCAATGAATATCAATTT
T.rangeli                --------------------------------------------------------------------------
T.conorhini              --------------------------------------------------------------------------
T.noyesi                 GGGGAGTTTTGGTCGT-AGGCGGGGTCGAGTT----CATCTCGCTCCTC-GTCTCGCCAATGAATATTAATTT
TrypAB2016a              GGGGAGTTTTGGTCGT-AGGCGGGGTCGAGTT----CATCTCGCTCCTC-GTCTCGCCAATGAATATTAATTT
TrypAB2013.G8            GGGGAGTTTTGGTCGT-AGGCAGG-TCGAGTT----CATCTCGCTCCTC-GTCTCGCCAATGAATATTAATTT
T.janseni                GGGGAGTTTTGGTCGT-AGGCGAGGTCGGGTT----CATCTCGCTCCTC-GTCTCGCCAATGAATATTAATTT
T.wauwau                 GGGGAGTTTTGGTCGC-AGGCGAGGTCGGGTT----CATCTCGCTCCTC-GTCTCGCCAATGAATATCAATTT
TrypAB2013.G7            GGGGAGTTTTGGTCTT-AGGC-----------------------------------------------------
TrypAB2013.G5            GGGGAGTTTTGGTCTT-AGGC-----------------------------------------------------
TrypAB2013.G4            GGGGAGTTTTGGTCTT-AGGC-----------------------------------------------------
TrypAB2013.G2            GGGGAGTTTTGGTCGC-AGGC-----------------------------------------------------
TrypAB2013.G1            GGGGAGTTTTGGTCGC-AGGC-----------------------------------------------------
T.lewisi                 GGGGAGTTTTGGTCGT-AGGCGGGGTCGGGTT----CATCTCGCTCCTC-GTCTCGCCAATGTATATCAATTT
T.microti                GGGGAGTTTTGGTCGT-AGGCGGGGTCGGGTT----CATCTCGCTCCTC-GTCTCGCCAATGTGTATCAATTT
T.livingstonei           GGGGAGTTTTGGTCGT-AGGCGTGGTCGGGTT----CATCCCGCTCCACCGCCTCGCCAATATATATCAATTT
Trypanosoma sp.wombat    GGGGAGTTTTGGTCGC-AGGC-----------------------------------------------------
Trypanosoma sp.bat       GGGGAGTTTTGGTCGTTAGACTAGGACGGGTT----CATCTCGTTTCTA-GTCTCGCCAATATTTATCAATTT
Trypanosoma sp.NanDoum   --------------------------------------------------------------------------
Trypanosoma sp.HochNDi   --------------------------------------------------------------------------
H.muscarum               GGGGAGTTTGGTTGCGCCCGGAGGCTTCCTTGTGAAGTCCTTCGGGCCACCGTTACTAATTTACGTGCAT-ATT
```

Fig. 1: alignment of 734-bp of 18S SSU sequences used in this study. Each species is represented here by one sequence. Grey area highlights the alignment region excluded in the phylogenetics analyses due to low-confidence homology. Conserved regions without gaps are marked with an asterisk (*).

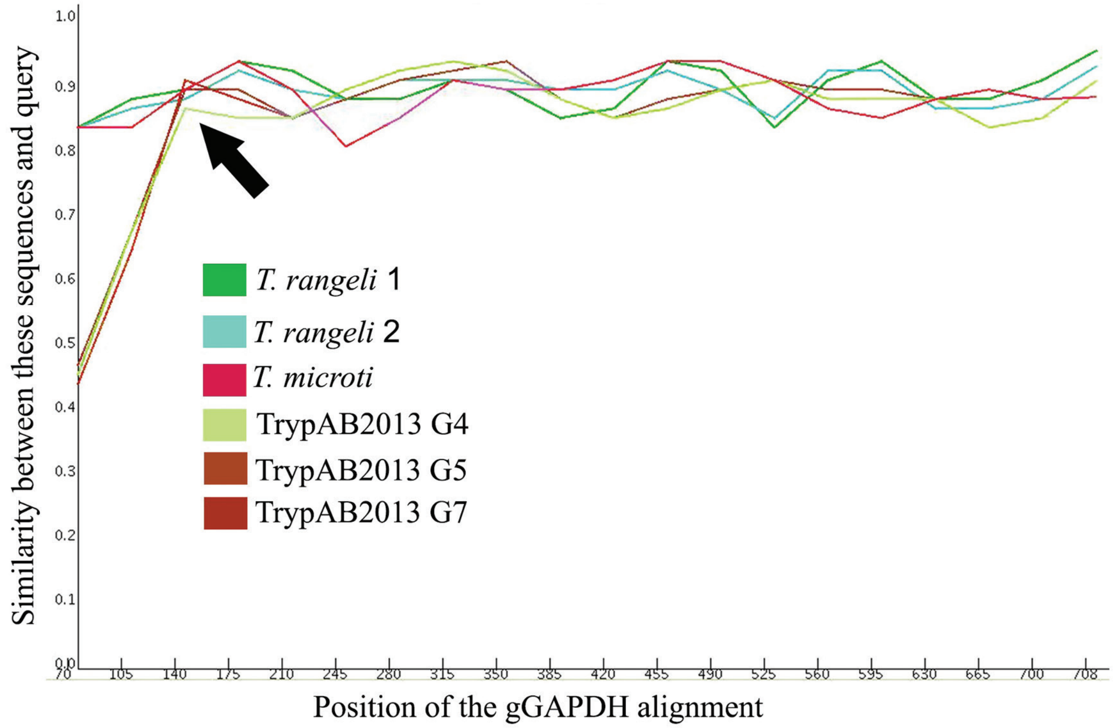

Fig. 2: recombination plot of six trypanosomatid samples of the 708-bp fragment of gGAPDH. Black arrow indicates the region with significant recombination ($p < 0.001$). Note that in this region there is a crossover point between *Trypanosoma rangeli* samples and the Australian AB2013 G5, G7 and *T. microti* sequences. Pairwise distances were calculated with the TrN model. In this example, *T. cruzi* was used as query.

REFERENCES

Borghesan TC, Ferreira RC, Takata CS, Campaner M, Borda CC, Paiva F, et al. Molecular phylogenetic redefinition of *Herpetomonas* (Kinetoplastea, Trypanosomatidae), a genus of insect parasites associated with flies. Protist. 2013; 164: 129-52.

Botero A, Cooper C, Thompson CK, Clode PL, Rose K, Thompson RC. Morphological and phylogenetic description of *Trypanosoma noyesi* sp. nov.: an Australian wildlife trypanosome within the *T. cruzi* clade. Protist. 2016; 167: 425-39.

Botero A, Thompson CK, Peacock CS, Clode PL, Nicholls PK, Wayne AF, et al. Trypanosomes genetic diversity, polyparasitism and the population decline of the critically endangered Australian marsupial, the brush tailed bettong or woylie (*Bettongia penicillata*). Int J Parasitol Parasites Wildl. 2013; 2: 77-89.

Cavazzana Jr M, Marcili A, Lima L, da Silva FM, Junqueira AC, Veludo HH, et al. Phylogeographical, ecological and biological patterns shown by nuclear (ssrRNA and gGAPDH) and mitochondrial (Cyt b) genes of trypanosomes of the subgenus *Schizotrypanum* parasitic in Brazilian bats. Int J Parasitol. 2010; 40 (3): 345-55.

da Costa AP, Nunes PH, Leite BH, Ferreira JI, Tonhosolo R, da Rosa AR, et al. Diversity of bats trypanosomes in hydroeletric area of Belo Monte in Brazilian Amazonia. Acta Trop. 2016; 164: 185-93.

Franzen O, Talavera-Lopez C, Ochaya S, Butler CE, Messenger LA, Lewis MD, et al. Comparative genomic analysis of human infective *Trypanosoma cruzi* lineages with the bat-restricted subspecies *T. cruzi marinkellei*. BMC Genomics. 2012; 13: 531.

Hamilton PB, Adams ER, Njiokou F, Gibson WC, Cuny G, Herder S. Phylogenetic analysis reveals the presence of the *Trypanosoma cruzi* clade in African terrestrial mammals. Infect Genet Evol. 2009; 9: 81-6.

Hamilton PB, Stevens JR, Gaunt MW, Gidley J, Gibson WC. Trypanosomes are monophyletic: evidence from genes for glyceraldehyde phosphate dehydrogenase and small subunit ribosomal RNA. Int J Parasitol. 2004; 34: 1393-404.

Hamilton PB, Stevens JR, Gidley J, Holz P, Gibson WC. A new lineage of trypanosomes from Australian vertebrates and terrestrial bloodsucking leeches (Haemadipsidae). Int J Parasitol. 2005; 35(4): 431-43.

Hamilton PB, Teixeira MM, Stevens JR. The evolution of *Trypanosoma cruzi*: the ‘bat seeding’ hypothesis. Trends Parasitol. 2012; 28: 136-41.

Lima L, Espinosa-Alvarez O, Hamilton PB, Neves L, Takata CS, Campaner M, et al. *Trypanosoma livingstonei*: a new species from African bats supports the bat seeding hypothesis for the *Trypanosoma cruzi* clade. Parasit Vectors. 2013; 6: 221.

Lima L, Espinosa-Alvarez O, Pinto CM, Cavazzana Jr M, Pavan AC, Carranza JC, et al. New insights into the evolution of the *Trypanosoma cruzi* clade provided by a new trypanosome species tightly linked to Neotropical *Pteronotus* bats and related to an Australian lineage of trypanosomes. Parasit Vectors. 2015; 8: 657.

Lima L, Silva FM, Neves L, Attias M, Takata CS, Campaner M, et al. Evolutionary insights from bat trypanosomes: morphological, developmental and phylogenetic evidence of a new species, *Trypanosoma* (*Schizotrypanum*) *erneyi* sp. nov., in African bats closely related to *Trypanosoma* (*Schizotrypanum*) *cruzi* and allied species. Protist. 2012; 163: 856-72.

da Silva FM, Junqueira AC, Campaner M, Rodrigues AC, Crisante G, et al. Comparative phylogeography of *Trypanosoma rangeli* and *Rhodnius* (Hemiptera: Reduviidae) supports a long coexistence of parasite lineages and their sympatric vectors. Mol Ecol. 2007; 16: 3361-73.

McInnes LM, Gillett A, Ryan UM, Austen J, Campbell RS, Hanger J, et al. *Trypanosoma irwini* n. sp (Sarcomastigophora: Trypanosomatidae) from the koala (*Phascolarctos cinereus*). Parasitology. 2009; 136: 875-85.

Paparini A, Irwin PJ, Warren K, McInnes LM, de Tores P, Ryan UM. Identification of novel trypanosome genotypes in native Australian marsupials. Vet Parasitol. 2011; 183: 21-30.

Pinto CM, Ocana-Mayorga S, Tapia EE, Lobos SE, Zurita AP, Aguirre-Villacís F, et al. Bats, trypanosomes, and triatomines in Ecuador: new insights into the diversity, transmission, and origins of *Trypanosoma cruzi* and Chagas disease. PLoS ONE. 2015; 10: e0139999.

Stevens J, Noyes H, Gibson W. The evolution of trypanosomes infecting humans and primates. Mem Inst Oswaldo Cruz. 1998; 93(5): 669-76.

Stevens JR, Teixeira MM, Bingle LE, Gibson WC. The taxonomic position and evolutionary relationships of *Trypanosoma rangeli*. Int J Parasitol. 1999; 29(5): 749-57.

Yurchenko V, Lukes J, Xu X, Maslov DA. An integrated morphological and molecular approach to a new species description in the Trypanosomatidae: the case of *Leptomonas podlipaevi* n. sp., a parasite of *Boisea rubrolineata* (Hemiptera: Rhopalidae). J Eukaryot Microbiol. 2006; 53 (2): 103-11.
